# Mutations in noncoding regions of *GJB1* are a major cause of X-linked CMT

**DOI:** 10.1212/WNL.0000000000003819

**Published:** 2017-04-11

**Authors:** Pedro J. Tomaselli, Alexander M. Rossor, Alejandro Horga, Zane Jaunmuktane, Aisling Carr, Paola Saveri, Giuseppe Piscosquito, Davide Pareyson, Matilde Laura, Julian C. Blake, Roy Poh, James Polke, Henry Houlden, Mary M. Reilly

**Affiliations:** From the MRC Centre for Neuromuscular Diseases (P.J.T., A.M.R., A.H., A.C., M.L., M.M.R.), Department of Neuropathology (Z.J.), and Department of Neurogenetics (R.P., J.P., H.H.), National Hospital for Neurology and Neurosurgery, UCL Institute of Neurology, Queen Square, London, UK; Clinic of Central and Peripheral Degenerative Neuropathies Unit (P.S., G.P., D.P.), Department of Clinical Neurosciences, IRCCS Foundation, C. Besta Neurological Institute, Milan, Italy; Department of Clinical Neurophysiology (J.C.B.), Norfolk and Norwich University Hospital, Norfolk, UK.

## Abstract

**Objective:**

To determine the prevalence and clinical and genetic characteristics of patients with X-linked Charcot-Marie-Tooth disease (CMT) due to mutations in noncoding regions of the gap junction β-1 gene (*GJB1*).

**Methods:**

Mutations were identified by bidirectional Sanger sequence analysis of the 595 bases of the upstream promoter region, and 25 bases of the 3′ untranslated region (UTR) sequence in patients in whom mutations in the coding region had been excluded. Clinical and neurophysiologic data were retrospectively collected.

**Results:**

Five mutations were detected in 25 individuals from 10 kindreds representing 11.4% of all cases of CMTX1 diagnosed in our neurogenetics laboratory between 1996 and 2016. Four pathogenic mutations, c.-17G>A, c.-17+1G>T, c.-103C>T, and c.-146-90_146-89insT were detected in the 5′UTR. A novel mutation, c.*15C>T, was detected in the 3′ UTR of *GJB1* in 2 unrelated families with CMTX1 and is the first pathogenic mutation in the 3′UTR of any myelin-associated CMT gene. Mutations segregated with the phenotype, were at sites predicted to be pathogenic, and were not present in the normal population.

**Conclusions:**

Mutations in noncoding DNA are a major cause of CMTX1 and highlight the importance of mutations in noncoding DNA in human disease. Next-generation sequencing platforms for use in inherited neuropathy should therefore include coverage of these regions.

Mutations in the gap junction β-1 gene (*GJB1*) encoding the transmembrane channel protein, connexin 32 (Cx32), are the most common cause of X-linked Charcot-Marie-Tooth disease (CMTX) and the second commonest cause of Charcot-Marie-Tooth disease (CMT) overall.^1^ The Cx32 protein is widely expressed in human tissues, including myelinating Schwann cells in the peripheral nervous system.^2^ In the peripheral nervous system, Cx32 is found in the noncompact myelin of the paranodes and incisures, where it allows the movement of small molecules and ions between the multiple concentric myelinated layers of the Schwann cell and the axon membrane.^2,3^ *GJB1* exists as 2 transcripts that are regulated by 2 tissue-specific promoters (P1 and P2), allowing differential expression of these transcripts in neuronal and non-neuronal tissue.^4–6^ The transcriptional machinery in neuronal tissue requires the P2 promoter and other elements located in the 5′ untranslated region (UTR) for efficient Cx32 expression. Mutations in the 5′ UTR region have previously been described by our group and others as causative of CMTX1 and have been shown to impair P2-mediated transcription of *GJB1*.^7^ Mutations in the 3′ UTR region are a rare cause of hereditary diseases overall; however, as this region often contains mRNA regulatory elements, mutations in the 3′ UTR may affect normal translation.^8^ In this study, we sought to determine the frequency and phenotype of CMTX1 due to mutations in the 5′ and 3′ UTR noncoding regions of *GJB1*.

## METHODS

### Patients.

Patients harboring mutations in the 5′ and 3′ UTR of *GJB1* were identified from the CMT database of the National Hospital for Neurology and Neurosurgery, Queen Square, London, United Kingdom. In some patients in whom Sanger sequencing of the coding region of *GJB1* was negative, further screening of the 5′ and 3′ UTR was performed because of the strong clinical suspicion of CMT1X on the basis of a lack of male-to-male transmission, more severely affected males, and a predominantly demyelinating polyneuropathy. The remaining patients were identified from diagnostic samples submitted for testing of *GJB1* to the neurogenetics laboratory of The National Hospital of Neurology and Neurosurgery after routine screening of both the coding and noncoding regions of *GJB1* was adopted. The clinical and neurophysiologic data were collected retrospectively for all identified patients with mutations in the 5′ and 3′ UTR of *GJB1*.

### Statistical analysis.

Statistical analysis was performed using a 2-tailed Student unpaired *t* test (Excel; Microsoft, Redmond, WA).

### Genetics analysis.

Genetic testing was performed in the National Hospital of Neurology and Neurosurgery Neurogenetics Laboratory. Additional targeted genetic testing was performed in selected cases (appendix e-1 at Neurology.org). Mutations were identified by bidirectional Sanger sequence analysis of *GJB1* including 595 bases upstream of the ATG start codon, the coding region, and 25 bases of 3′ UTR sequence. Conditions and primers are available in appendix e-1. In silico analysis was performed with the aid of AlamutVisual (Interactive Biosoftware, Rouen, France), which includes the splice-prediction tools SpliceSiteFinder-like, MaxEntScan, NNSPLICE, GeneSplicer, and Human Splicing Finder.

### Standard protocol approvals, registrations, and patient consents.

This study was approved by the research ethics committee of the National Hospital for Neurology and Neurosurgery. All patients consented to publication of their clinical details.

## RESULTS

### Demographics.

**Figure 1:**
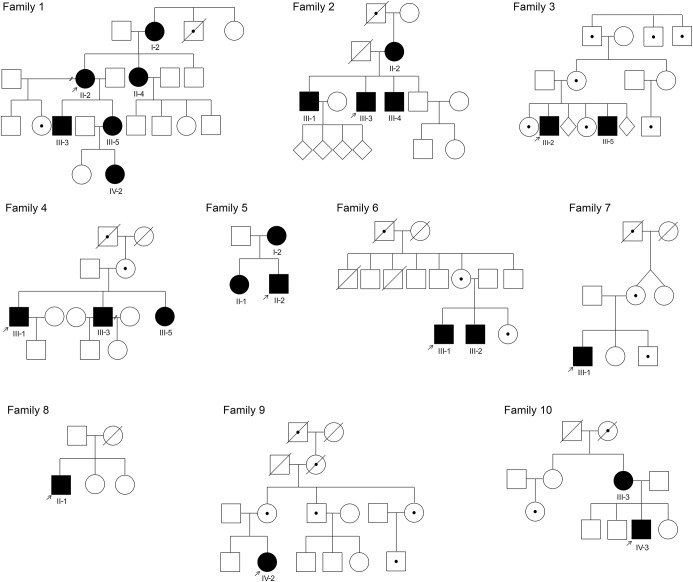
Pedigrees for the families reported in this study Black symbols = affected; empty symbols = unaffected; dot symbols = affected by history; diagonal line = deceased; arrow = index case.

A total of 25 individuals from 10 kindreds with mutations in the 5′ and 3′ UTR of *GJB1* were identified (figure 1), of whom 14 were male and 11 female. The age at onset was reported to be less than 10 years in 7 male participants and 1 female participant with a range of 5–32 years in male participants and 8–55 years in female participants. Four patients from family 1 (1-I.2, 1-II.2, 1-II.4, and 1-III.3) and all 4 patients from family 2 have been reported previously.^9^ There was no male-to-male transmission in any of the pedigrees. From 1996 to 2016, 194 patients with mutations in the open reading frame of *GJB1* were identified. Mutations in the 5′ and 3′ UTR therefore represent 11.4% of patients with CMTX1 identified in our neurogenetics laboratory.

### Clinical features.

The clinical details of the cohort are summarized in table 1. The most common presenting complaint was difficulty walking. Male participants were more severely affected than female participants. The mean Charcot-Marie-Tooth Examination Score was 6.30 ± 5.31 (range 0–14, n = 10) for female participants and 11.5 ± 3.81 (range 7–18, n = 10), for male participants (*p* = 0.021). One woman (2-II.2, aged 58) harboring the c.-17G>A mutation and 3 women (5-I.2, 5-II.1, and 10-III.3, aged 58, 29, and 71, respectively) harboring the c.*15C>T mutation were asymptomatic but all had abnormal nerve conduction studies (table 2). Atypical presentations in our cohort included the following: patient 1-I.2 from family 1 (c.-17G>A) presented with late-onset CMTX (age 55), unilateral deafness, and Horner syndrome, characterized by miosis and ptosis. The proband (1-II.2) had mild scoliosis and her son (1-III.3) presented at age 8 years with hand tremor and difficulty writing. Three patients (1-I.2, 2-III.3, and 4-III.3) from families 1, 2, and 4 harboring the c.-17G>A mutation had unilateral extensor plantar responses. Postural tremor was present in 5 out of 6 male participants and 2 out of 7 female participants harboring the c.-17G>A mutation. Twelve patients had a split-hand, characterized by disproportionate involvement of the abductor pollicis brevis compared with the first dorsal interosseous and abductor digiti minimi muscles.

**Table 1:**
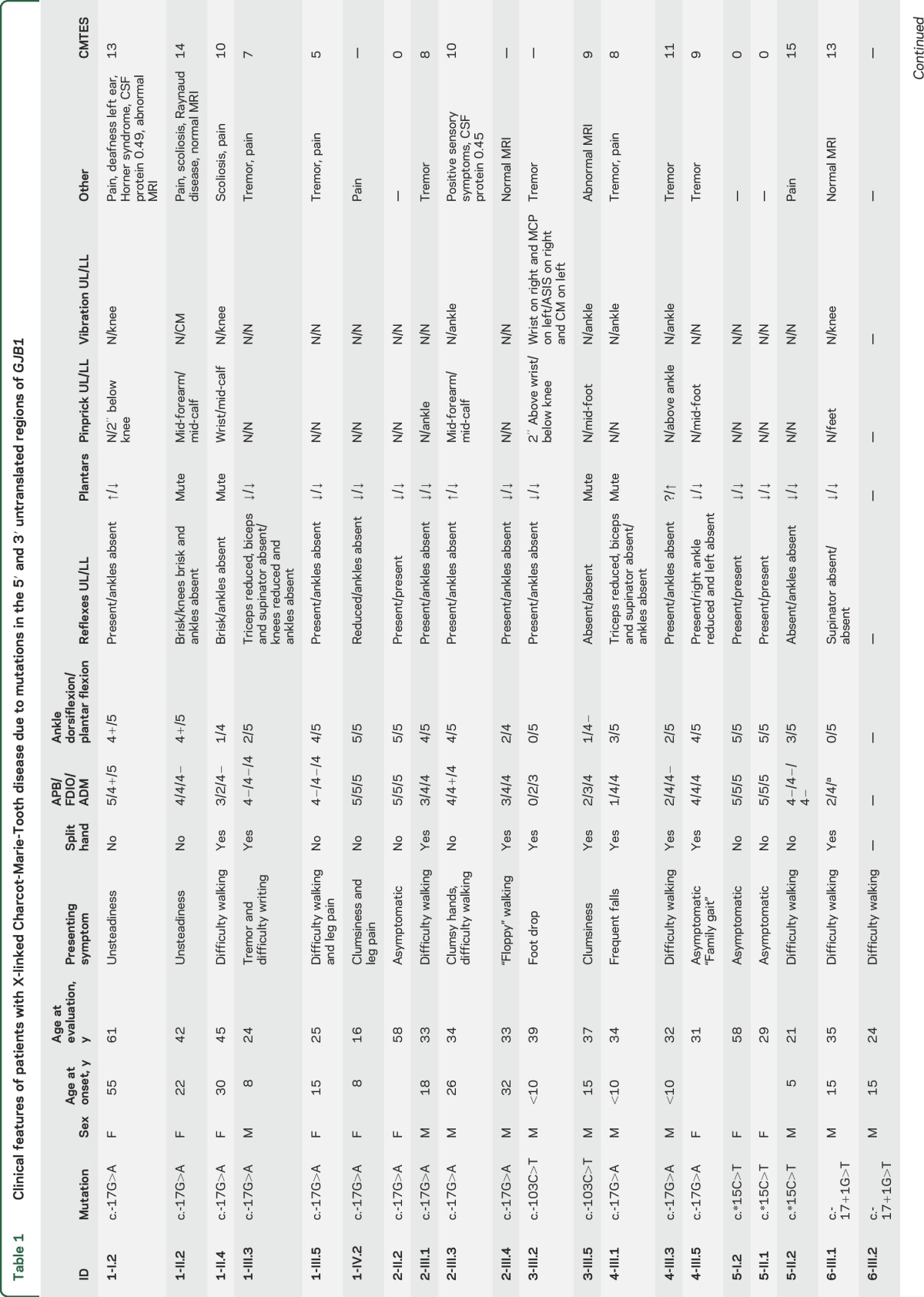

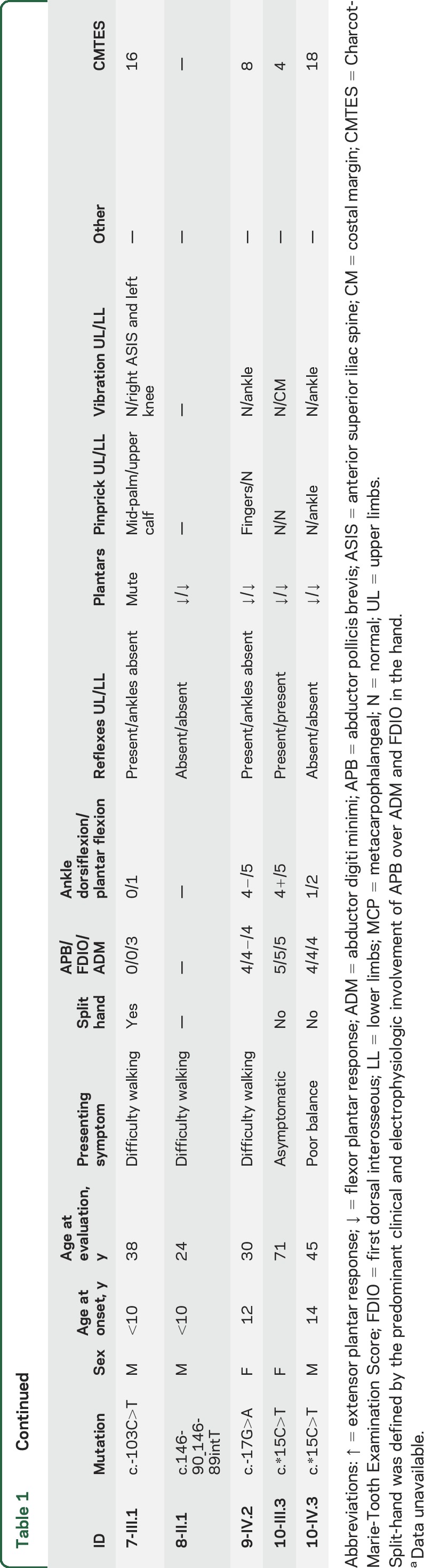
Clinical features of patients with X-linked Charcot-Marie-Tooth disease due to mutations in the 5′ and 3′ untranslated regions of *GJB1*

**Table 2:**
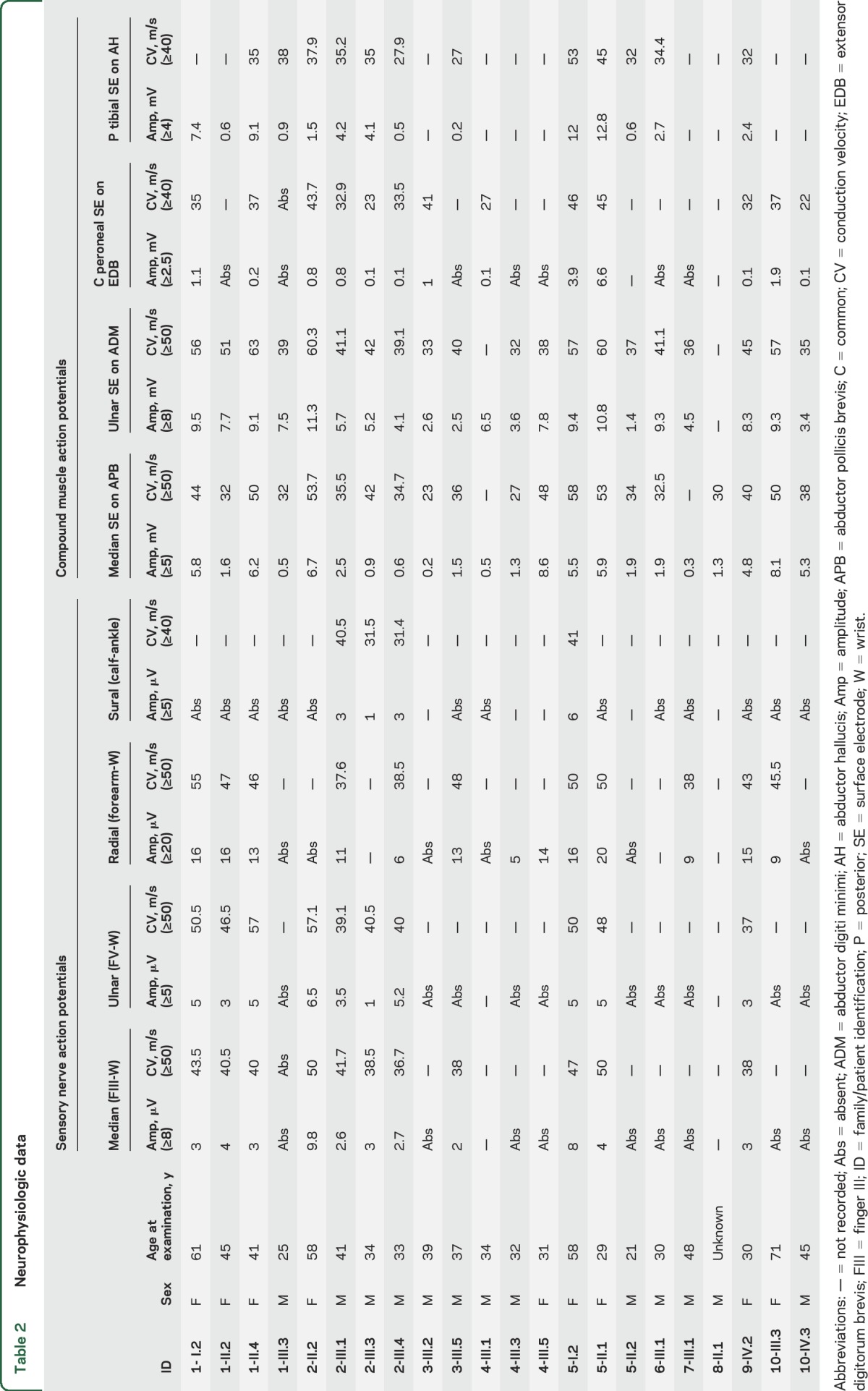
Neurophysiologic data

### Neurophysiology.

Nerve conduction studies were available in 22 individuals (13 male and 9 female) and in all cases demonstrated a motor and sensory neuropathy (table 2). In male participants, the mean ulnar motor nerve conduction velocity (CV) was 37.78 ± 3.43 m/s (range 32–42 m/s), whereas in female participants, the mean ulnar CV was 54.15 ± 8.09 m/s (range 38–63 m/s), *p* < 0.0001 (tables 2 and e-1). There was a discrepancy between the median and ulnar compound muscle action potentials' amplitude, with the former being significantly reduced compared to the latter (table e-1). This finding is in accordance with the clinical observation of the split-hand.^10^

### MRI.

Brain MRI was performed in 5 patients (3 male and 2 female) and revealed a lesion of the corpus callosum in 1 female patient (1-I.2) with no vascular risk factors or clinical features suggestive of multiple sclerosis (appendix e-1).

### Neuropathology.

Sural nerve biopsy was available from 2 patients (1-II.2 and 2-III.3) and revealed a significant reduction in myelinated nerve fiber density and thin myelin sheaths (appendix e-1). There were occasional regeneration clusters and mild endoneurial edema. There were no inflammatory cells. These findings are similar to coding *GJB1* mutation patients.

### Genetic analysis.

**Figure 2:**
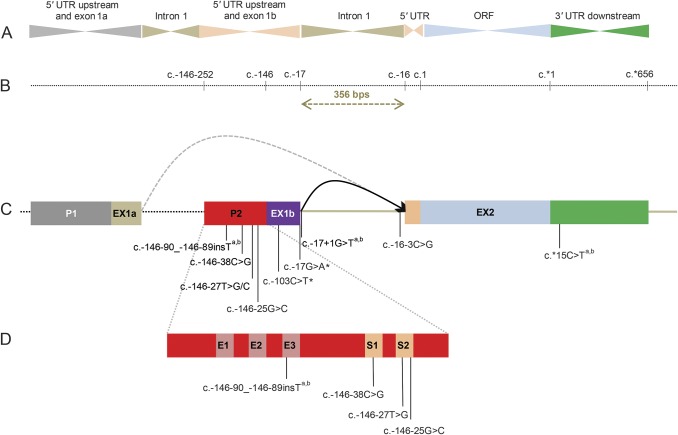
*GJB1* gene structure with mutations in noncoding regions highlighted (A) Structural organization of *GJB1*. (B) Base numbering at each junction between regions according to the Human Genome Variation Society. (C) *GJB1* has 2 tissue-specific promoters (P1 and P2) that are alternatively spliced. In liver and pancreas, *GJB1* transcription is driven via promoter 1 (P1) upstream of the noncoding exon, exon 1a, whereas in neural tissue it is driven via the nerve-specific promoter 2 (P2) upstream of noncoding exon 1b.^4,6^ The P1- and P2-expressed mRNAs have different 5′ untranslated regions (UTRs) but an identical open reading frame (ORF) region and 3′ UTR. (D) The EGR2 (E1, E2, and E3) and SOX10 (S1 and S2) binding sites of the P2 promoter region that function synergistically to regulate Cx32 expression in the nervous system. ^a^ Variants included in this study. ^b^ Novel variants.

Five distinct mutations in the 5′ and 3′ UTR of *GJB1* were identified (table 1). The position of the mutations relative to the *GJB1* open reading frame (ORF) region is shown in figure 2. The nomenclature used in this study is based on current recommendations of the Human Genome Variation Society (HGVS).^11^ In table e-2, there is an overview of all mutations in the 5′ and 3′ UTR regions of *GJB1* and the corresponding nomenclature based on counting directly from the ATG translation initiation codon, which has been previously used to describe a number of mutations. The previously reported mutations^9,12–15^ c.-103C>T and c.-17G>A were detected in 2 (3 and 7) and 4 unrelated families (1, 2, 4, and 9), respectively, and segregated with the phenotype in all family members tested. Three novel mutations were identified: c.-146-90_-146-89insT in family 8, c.-17+1G>T in family 6, and c.*15C>T in families 5 and 10. The genome conservation scores are shown in table e-3; they were assessed using PhiloP, which were accessed through the UCSC Genome Browser (GRCh37/hg19).^16^ These 3 novel mutations segregate with the phenotype and are predicted to be pathogenic using AlamutVisual (Interactive Biosoftware) software. They are not present in the NHLBI Exome Sequencing Project (EVS), Exome Aggregation Consortium (ExAC), dbSNP, or the 1000 Genome (1000genomes) databases.^17–20^ ExAC and EVS only include the ORF and ±50 bp of intronic sequences. The 2 new variants within the 5′ UTR region were not detected in 100 controls. The new variant in the 3′ UTR is not present in ExAC. The scores of the in silico splicing analysis for c.*15C>T are shown in table e-4.

## DISCUSSION

In this study, we describe 2 new pathogenic mutations in the 5′ UTR and a likely pathogenic mutation in the downstream 3′ UTR region of *GJB1*. The evidence for the pathogenicity of these mutations is largely indirect and based on a typical CMT X1 phenotype, segregation within family members, in silico splice prediction analysis, and for the 3′ UTR mutation, the presence of the same mutation in an unrelated individual with the same phenotype. All patients included in this study had a clinical or neurophysiologic phenotype typical for CMTX1 due to mutations in the *GJB1* ORF region, characterized by a slowly progressive, predominantly length-dependent neuropathy, in which male participants were more severely affected than female participants and with an earlier age at onset.^21^ In male participants, the motor CVs were in the intermediate range and slower than in female participants, as has been described previously.^22^ Evidence suggests that loss of Cx32 channel function is the underlying pathomechanism responsible for CMTX1 due to coding mutations in *GJB1*.^23^

The nerve-specific 5′ UTR of *GJB1* is located immediately upstream of the start codon, adjacent to the P2 promoter. The P2 promoter contains binding sites for the neuron-specific transcription factors SOX10 and EGR2 that strongly activate Cx32 expression in the peripheral nervous system.^7,24^ EGR2 has 3 proposed binding sites (E1, E2, and E3) within the P2 promoter, whereas SOX10 has 2 P2 binding sites (S1 and S2) (figure 2). Several mutations located within the core of the S2 SOX10 binding site have previously been described, a number of which have been shown to impair SOX10-mediated transcription of *GJB1,* resulting in a significant reduction in Cx32 expression.^7,12,25,26^ The novel c.-146-90_-146-89insT mutation is located within the E3 *EGR2* binding site. The E2 and E3 binding sites of promoter P2 are responsible for the majority of EGR2-mediated transcriptions of *GJB1*.^24^ It is therefore likely that this mutation results in reduced Cx32 expression as observed for mutations within the SOX10 binding site.

The c.-17G>A mutation was identified in 4 different families (1, 2, 4, and 9) and has previously been reported by our group.^9^ This mutation is located in the last base of exon 1b, which is one of the most highly conserved bases in splice-site consensus sequences. In silico splice site analysis predicted that this mutation may reduce the efficiency of splicing at this intron/exon boundary, leading to the inclusion of intron 1 and a mutant transcript.^9^ The second novel c.-17+1G>T mutation in family 6 affects the adjacent base and is predicted to be pathogenic by the same mechanism.

We identified the c.-103C>T mutation in 2 different families (3 and 7). This mutation has previously been reported in unrelated families.^12,14,15,27^ It is located within exon Ib, downstream of the P2 promoter, and lies within the internal ribosomal entry site (IRES) of the peripheral nerve specific mRNA transcript. The mutation is predicted to prevent translation of *GJB1* mRNA.^28^ Taken together, our study and previous reports provide strong evidence that the c.-103C>T is pathogenic. Of note, the c.-102G>A variant (reported as c.-458G>A), affecting the adjacent base, did not segregate in a large family with CMT, suggesting that not every variant of an IRES element is pathogenic.^29^

Despite being located in a less conserved region, the mutation in the 3′ UTR region, c.15C>T*, segregated in 2 unrelated families with a typical CMTX1 phenotype. This specific 3′ UTR region contains sequences that are predicted to act as regulatory elements critical in Cx32 translational activation/repression, mRNA stability, micro-RNA binding, and transcript localization.^8^ Although our understanding of the role of these sequences is poor, in silico splicing analysis predicts that this variant may create a 5′ donor splice site leading to aberrant splicing within the 3′ UTR. This in turn may affect mRNA stability, leading to downregulation of GJB1 expression.

The clinical and electrophysiologic findings of the patients described in this article with point mutations within S2 SOX10 and E3 EGR2 are indistinguishable from patients with mutations in the ORF of *GJB1*. Previous in vitro analysis of the c.-146-27T>C (c.-529T>C) mutation in the E3 region or deletion of the S2 region have demonstrated a partial loss of promoter activity.^7,24^ The indistinguishable clinical phenotype of the patients included in this study from patients with complete loss of function mutations in *GJB1* suggests that the noncoding mutations described cause complete loss of function.

In this study, we describe 5 pathogenic mutations, 3 of which are novel, in noncoding regions of *GJB1*, which are predicted to result in loss of function by a combination of transcription factor binding, disruption of mRNA translation, and altered mRNA stability. The search for these noncoding mutations was largely driven by the recognition of the classical phenotype of CMTX1 in the absence of mutations in the coding regions of *GJB1*. The large number of noncoding mutations in *GJB1* (11.4% of our cohort of 219 *GJB1* patients) is of interest and highlights the importance of mutations in noncoding DNA in human disease and the need to include noncoding regions of *GJB1* in targeted inherited neuropathy gene panels.

The study also raises the possibility that these types of mutations may be a more frequent cause of other inherited neurologic conditions than has been previously appreciated including in the not infrequent situation where next-generation sequencing identifies a heterozygous mutation for a gene known to cause recessive disease. How frequent similar noncoding mutations will be in other hereditary neuropathies and other inherited neurologic diseases has yet to be determined.

## Supplementary Material

Data Supplement

## GLOSSARY

CMT: Charcot-Marie-Tooth disease
CMTX: X-linked Charcot-Marie-Tooth disease
CV: conduction velocity
Cx32: connexin 32
EVS: Exome Sequencing Project
ExAC: Exome Aggregation Consortium
*GJB1*: gap junction β-1 gene
IRES: internal ribosomal entry site
ORF: open reading frame
UTR: untranslated region
