# Supplementary material for: Mutations in noncoding regions of GJB1 are a major cause of X-linked CMT
Source: Neurology. 2017 Apr 11;88(15):1445–53. doi: 10.1212/WNL.0000000000003819 (PMC5386440; doi:10.1212/WNL.0000000000003819)
Supplement: Data Supplement [file supp_88_15_1445__index.html]

Mutations in noncoding regions of GJB1 are a major cause of X-linked CMT — Data Supplement 

# Mutations in noncoding regions of *GJB1* are a major cause of X-linked CMT

## Data Supplement

**Neurology® data supplements are not copyedited before publication. Published editorials and translations have been copyedited.  
 © 2017 American Academy of Neurology.  
  
 Files in this Data Supplement:**

- Appendix e-1 - PDF
- Table e-1 - PDF
- Table e-2 - PDF
- Table e-3 - PDF
- Table e-4 - PDF
