# Supplementary material for: Mutations in noncoding regions of GJB1 are a major cause of X-linked CMT
Source: Neurology. 2017 Apr 11;88(15):1445–53. doi: 10.1212/WNL.0000000000003819 (PMC5386440; doi:10.1212/WNL.0000000000003819)
Supplement: Data Supplement [file supp_WNL.0000000000003819_Appendix_e-1.pdf]

Appendix e-1

Sanger sequencing. The upstream non-coding sequence, coding sequence and 25 bp of 3'UTR of *GJB1* were amplified using four overlapping M13-tailed primer pairs listed in the table below. PCRs were performed with AmpliTaq Gold 360 Master Mix (Applied Biosystems, Foster City, California, USA), with an annealing temperature of 58ºC. Sequencing reactions were performed using M13 primers and BigDye Terminator cycle sequencing chemistry (Applied Biosystems, Foster City, California, USA). Sequences were analysed on an ABI3730XL automated DNA sequencer and SeqScape software (Applied Biosystems, Foster City, California, USA).

Primer Pair Name	FWD Sequence	RVS Sequence
**GJB1_Fg1**	TGGGACACAAGTGCTCTGTG	ACTCTTCCAAGAGCCAGTAG
**GJB1_Fg2**	GAGGTCAAGTCAAGTGAGTG	TTCACCTCCTCCAGGTGTAG
**GJB1_Fg3**	CTGCAGCTCATCCTAGTTTC	ATGTCTTTCAGGGAGCCATC
**GJB1_Fg4**	AGGTGGTGTACCTCATCATC	CCATCTCTTGTACCCAGTGG

All families included in this study underwent negative screening of the open reading frame of *GJB1*. In addition, the following genetic tests were performed and were negative.

Ch17=chromosome 17, PMP22=peripheral myelin protein 22, GDAP1=ganglioside-induced differentiation associated protein 1, NEFL=neurofilament light chain.

Family	Genes tested
**Family 1**	ch17 deletion/duplication; PMP22 sequencing, MPZ, GDAP1
**Family 2**	ch17 deletion/duplication; PMP22 sequencing, MPZ
**Family 3**	ch17 deletion/duplication
**Family 4**	ch17 deletion/duplication, MPZ
**Family 5**	-
**Family 6**	ch17 deletion/duplication, MPZ
**Family 7**	-
**Family 8**	ch17 deletion/duplication, MPZ
**Family 9**	-
**Family 10**	ch17 deletion/duplication, PMP22 sequencing, MPZ, NEFL

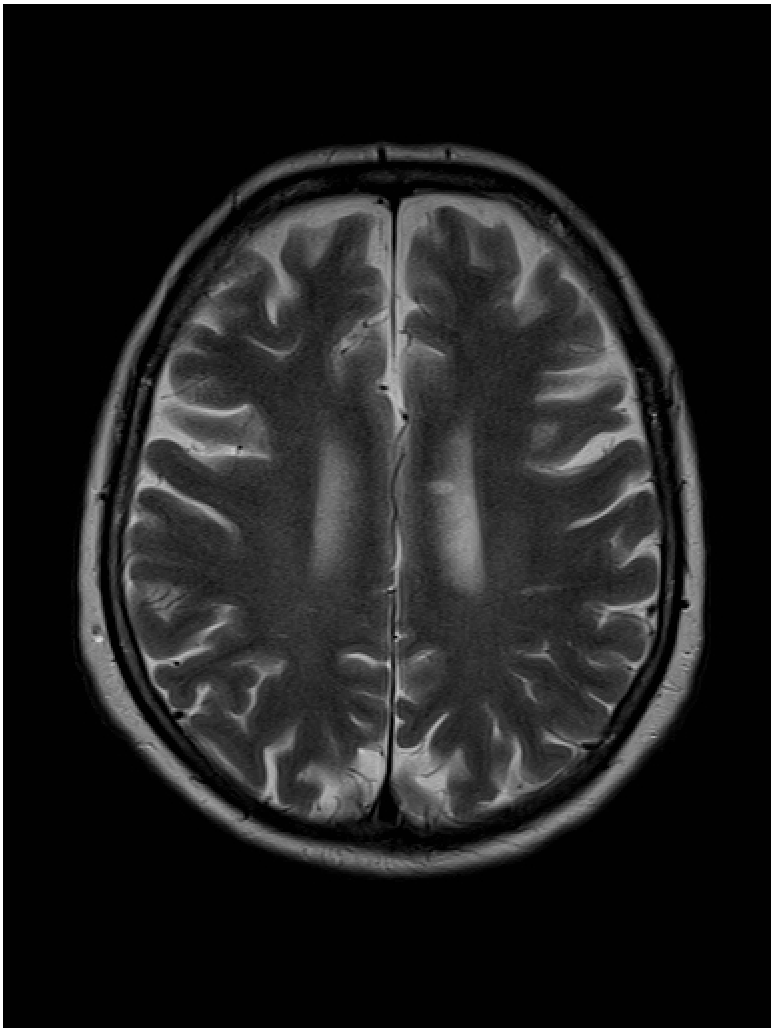

Brain MRI. The brain MRI of patient 1-I.2 demonstrating a lesion of the left corpus callosum

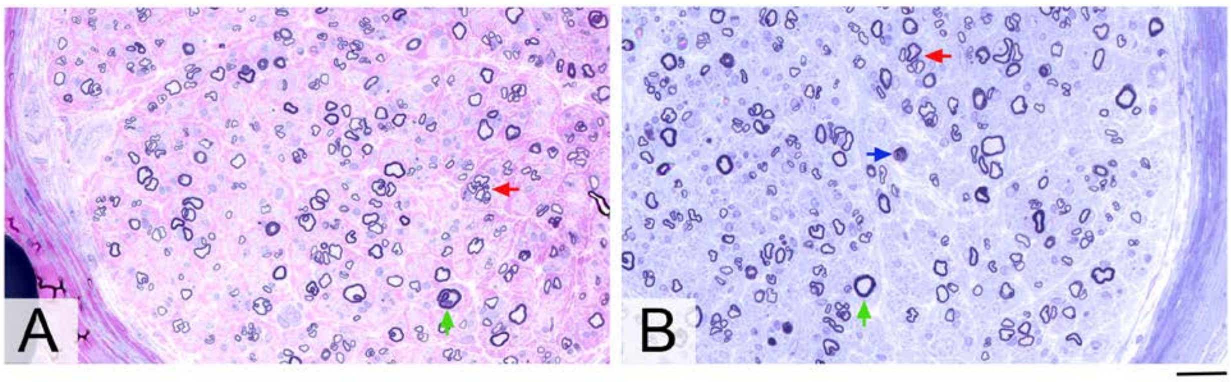

Morphological appearances of sural nerve biopsies from two patients with mutations in the non-coding regions of *GJB1*. Image A is from Case 3 and image B is from Case 1. Semi-thin resin preparations stained with methylene blue azure – basic fuchsin (A) and toluidine blue (B) show transverse sections of nerve fascicles with prominent loss of large normally myelinated fibers (green arrows in A and B) and frequent regenerating fibres (red arrows in A and B). The overall fiber density is mildly reduced. Actively degenerating axons are rare (blue arrow in figure B). There are no onion bulb formations to suggest chronic demyelination. Scale bar: 40µm in A and B
