# Supplementary material for: Mutations in noncoding regions of GJB1 are a major cause of X-linked CMT
Source: Neurology. 2017 Apr 11;88(15):1445–53. doi: 10.1212/WNL.0000000000003819 (PMC5386440; doi:10.1212/WNL.0000000000003819)
Supplement: Data Supplement [file supp_WNL.0000000000003819_Table_e-1.pdf]

Supplementary Table e-1. Comparison of electrophysiological parameters of median and ulnar nerve, male and females.

	**Ulnar CV (m/s)**			**CMAPs amplitude (mV) All patients**			**CMAP amplitude (mV) Female**			**CMAP amplitude (mV) Male**	
	**Male**	**Female**		**Median**	**Ulnar**		**Median**	**Ulnar**		**Median**	**Ulnar**
**Mean ± SD (n)**	38.03 ± 3.45 (10)	53.79 ± 8.58 (8)		2.92 ± 2.63 (20)	6.67 ± 2.96 (19)		5.64 ± 1.98 (8)	9.24 ± 1.32 (8)		1.12 ± 0.73 (12)	4.81 ± 2.35 (11)
**p-value**	<0.0001			0.0002			0.0008			<0.0001	
