# Supplementary material for: Mutations in noncoding regions of GJB1 are a major cause of X-linked CMT
Source: Neurology. 2017 Apr 11;88(15):1445–53. doi: 10.1212/WNL.0000000000003819 (PMC5386440; doi:10.1212/WNL.0000000000003819)
Supplement: Data Supplement [file supp_WNL.0000000000003819_Table_e-2.pdf]

Supplementary Table e-2. Overview of mutations identified in the 5' and 3' UTR regions of *GJB1*, including previously reported mutations. HGVS = Human Genome Variation Society; IRES = Internal Ribosomal Entry Site.

Position	Position relative to ATG	Current HGVS Nomenclature	Proposed Pathomechanism	Reference
Upstream P2	c.-592_-591insT	c.-146-90_-146-89insT	Impaired EGR2 mediated transcription	This paper
Upstream P2	c.-540C>G	c.-146-38C>G	None proposed	Silvera et al. [1]
Upstream P2	c.-529T>G	c.-146-27T>G	Impaired SOX10 mediated transcription	Ioanasescu *et al.* [2]
Upstream P2	c.-529T>C	c.-146-27T>C	Impaired SOX10 mediated transcription	Beauvais *et al.* and Tsai *et al.* [3, 4]
Upstream P2	c.-527G>C	c.-146-25G>C	Impaired SOX10 mediated transcription	Houlden *et al.* [5]
Exon 1 (5'UTR)	c.-459C>T	c.-103C>T	IRES mediated translation	Ioanasescu et al., Tsai et al., Li et al., Kabzinska et al. [2, 4, 6, 7] This paper
Exon 1 (5'UTR)	c.-373G>A	c.-17G>A	Aberrant splicing	Murphy *et al.* [8] This paper
Intron 1	c.-372G>T	c.-17+1G>T	Aberrant splicing	This paper
Intron 1	c.-19C>G	c.-16-3C>G	Aberrant splicing	Benedetti *et al.* [9]

Exon2 (3’UTR)	c.876C>T	c.*15C>T	Altered mRNA stability	This paper

References

1. Sivera R, Sevilla T, Vilchez JJ, Martinez-Rubio D, Chumillas MJ, Vazquez JF, Muelas N, Bataller L, Millan JM, Palau F, Espinos C. Charcot-Marie-Tooth disease: Genetic and clinical spectrum in a Spanish clinical series. Neurology 2013; 81, 1617-1625.
2. Ionasescu VV, Searby C, Ionasescu R, Neuhaus IM, Werner R. Mutations of the noncoding region of the connexin32 gene in X-linked dominant Charcot-Marie-Tooth neuropathy. Neurology 1996;47:541-544.
3. Beauvais K, Furby A, Latour P. Clinical, electrophysiological and molecular genetic studies in a family with X-linked dominant Charcot-Marie-Tooth neuropathy presenting a novel mutation in GJB1 Promoter and a rare polymorphism in LITAF/SIMPLE. Neuromuscul Disord 2006;16:14-18.
4. Tsai PC, Chen CH, Liu AB, et al. Mutational analysis of the 5' non-coding region of GJB1 in a Taiwanese cohort with Charcot-Marie-Tooth neuropathy. J Neurol Sci 2013;332:51-55.
5. Houlden H, Girard M, Cockerell C, et al. Connexin 32 promoter P2 mutations: a mechanism of peripheral nerve dysfunction. Ann Neurol 2004;56:730-734.
6. Li M, Cheng TS, Ho PW, et al. -459C>T point mutation in 5' non-coding region of human GJB1 gene is linked to X-linked Charcot-Marie-Tooth neuropathy. J Peripher Nerv Syst 2009;14:14-21.

7. Kabzinska D, Kotruchow K, Ryniewicz B, Kochanski A. Two pathogenic mutations located within the 5'-regulatory sequence of the GJB1 gene affecting initiation of transcription and translation. Acta Biochim Pol 2011;58:359-363.

8. Murphy SM, Polke J, Manji H, et al. A novel mutation in the nerve-specific 5'UTR of the GJB1 gene causes X-linked Charcot-Marie-Tooth disease. J Peripher Nerv Syst 2011;16:65-70.

9. Benedetti S, Previtali SC, Coviello S, et al. Analyzing histopathological features of rare charcot-marie-tooth neuropathies to unravel their pathogenesis. Arch Neurol 2010;67:1498-1505.
