# Supplementary material for: Mutations in noncoding regions of GJB1 are a major cause of X-linked CMT
Source: Neurology. 2017 Apr 11;88(15):1445–53. doi: 10.1212/WNL.0000000000003819 (PMC5386440; doi:10.1212/WNL.0000000000003819)
Supplement: Data Supplement [file supp_WNL.0000000000003819_Table_e-3.pdf]

**Supplementary Table e-3.** Genome conservation scores for each mutated nucleotide already reported within non-coding regions of *GJB1*.

| **Mutation** | **phastCons** | **phyloP** |
|---|---|---|
| c.-146-90_-146-89insT | 1 | 4.81 |
| c.-146-38C>G | 1 | 5.35 |
| c.-146-27T>G | 1 | 6.58 |
| c.-146-27T>C | 1 | 6.58 |
| c.-146-25G>C | 1 | 8.35 |
| c.-103C>T | 1 | 4.45 |
| c.-17G>A | 1 | 4.45 |
| c.-17+1G>T | 1 | 6.34 |
| c.-16-3C>G | 1 | 2.8 |
| c.*15C>T | 0.24 | 0.44 |
