# Supplementary material for: Mutations in noncoding regions of GJB1 are a major cause of X-linked CMT
Source: Neurology. 2017 Apr 11;88(15):1445–53. doi: 10.1212/WNL.0000000000003819 (PMC5386440; doi:10.1212/WNL.0000000000003819)
Supplement: Data Supplement [file supp_WNL.0000000000003819_Table_e-4.pdf]

Supplementary Table e-4. *In-silico* splicing analyses for the c.*15C>T mutation. This table shows the scores of the *in-silico* analysis performed using AlamutVisual® (Interactive Biosoftware Version 2.7.1) using the following splice-prediction: SpliceSiteFinder-like (SSF), MaxEntScan (MaxEnt), NNSPLICE, GeneSplicer and Human Splicing Finder (HSF). Each tool has its own score range, indicated in brackets under the tool name in the first row. The scores are shown as 'Wild-Type sequence score→Mutant sequence score'. The c.*15C>T mutation is strongly predicted to create a donor splice site at position c.*13 which may lead to aberrant GJB1 expression.

			**SSF (0-100)**	**MaxEnt (0-12)**	**NNSPLICE (0-1)**	**GeneSplicer (0-15)**	**HSF (0-100)**
**Donor site**	c.*15C>T	Exon 2 – c.*13	79.04→81.53	- →8.66	-→0.89	-→11.84	- →85.81
